# The role of Foxo3a in neuron-mediated cognitive impairment

**DOI:** 10.3389/fnmol.2024.1424561

**Published:** 2024-06-19

**Authors:** Qin-Qin Liu, Gui-Hua Wu, Xiao-Chun Wang, Xiao-Wen Xiong, Rui-Wang, Bao-Le Yao

**Affiliations:** Department of Rehabilitation Medicine, Ganzhou People’s Hospital, Ganzhou, China

**Keywords:** cognitive impairment, neurons, Foxo3a, cognitively impaired diseases, brain

## Abstract

Cognitive impairment (COI) is a prevalent complication across a spectrum of brain disorders, underpinned by intricate mechanisms yet to be fully elucidated. Neurons, the principal cell population of the nervous system, orchestrate cognitive processes and govern cognitive balance. Extensive inquiry has spotlighted the involvement of Foxo3a in COI. The regulatory cascade of Foxo3a transactivation implicates multiple downstream signaling pathways encompassing mitochondrial function, oxidative stress, autophagy, and apoptosis, collectively affecting neuronal activity. Notably, the expression and activity profile of neuronal Foxo3a are subject to modulation via various modalities, including methylation of promoter, phosphorylation and acetylation of protein. Furthermore, upstream pathways such as PI3K/AKT, the SIRT family, and diverse micro-RNAs intricately interface with Foxo3a, engendering alterations in neuronal function. Through several downstream routes, Foxo3a regulates neuronal dynamics, thereby modulating the onset or amelioration of COI in Alzheimer’s disease, stroke, ischemic brain injury, Parkinson’s disease, and traumatic brain injury. Foxo3a is a potential therapeutic cognitive target, and clinical drugs or multiple small molecules have been preliminarily shown to have cognitive-enhancing effects that indirectly affect Foxo3a. Particularly noteworthy are multiple randomized, controlled, placebo clinical trials illustrating the significant cognitive enhancement achievable through autophagy modulation. Here, we discussed the role of Foxo3a in neuron-mediated COI and common cognitively impaired diseases.

## 1 Introduction

Cognitive impairment (COI) is a brain disorder involved in impaired memory, language, reasoning, calculation, executive ability, and attention. Conditions such as Alzheimer’s disease (AD), stroke, ischemic brain injury (IBI), Parkinson (PA), and traumatic brain injury (TBI) are representative examples of COI disorders. Persistent COI seriously affects the recovery of patients’ ability to live autonomously. The development of COI is closely associated with damage in specific brain regions such as the hippocampus (Lisman et al., 2017), dorsolateral prefrontal cortex (Joyce et al., 2024), lateral amygdala (Qin et al., 2024), and pyriform cortex (Qin et al., 2024). In the field of brain rehabilitation therapy in recent years, the restoration of cognitive function has garnered significant recognition as a fundamental aspect of recovering brain functionality. Nevertheless, cognition encompasses a vast array of disorders with intricate etiology, and the precise underlying causes of COI remain incompletely explicated at present.

The core cause of COI pathogenesis at the cellular level involves massive neuronal death and dysfunction (Balakrishnan et al., 2024; Li et al., 2024). Neurons, the predominant cellular entities within the nervous system, establish synaptic connections with one another to form intricate neural circuits, critical for regulating behavioral development and consciousness (Cohen and Greenberg, 2008; Dow-Edwards et al., 2019; Südhof, 2021). Synapses are specialized adhesions for intercellular communication, either chemical or electrical (Miller et al., 2015; Nagy et al., 2018). Chemical synapses rely on various neurotransmitters, including dopamine and epinephrine, etc., to convey signals between neurons (Bito, 2010). Electrical synapses facilitate interneuronal transmission via direct ionic and metabolic coupling facilitated by gap junctions (Shimizu and Stopfer, 2013). Furthermore, interneuronal connections exhibit plasticity, which exert influence over the strength and durability of interneuronal communication, thereby modulating various facets of cognition (Amtul, 2015). Notably, neuronal survival has emerged as a pivotal endogenous factor implicated in the onset of numerous brain disorders, such as AD, stroke, PA, TBI, etc, contributing to the generation of COI. Thus, elucidating the modulation of neuronal function represents a crucial breakthrough in the amelioration of COI.

Forkhead box O3 (Foxo3a), also known as Foxo3, is a member of the Foxo family of transcription factors, which includes Foxo1, Foxo2, Foxo3, and Foxo4. Among these, Foxo3a has been the most extensively studied and recognized. Recent research has delved deeply into the role of Foxo3a in brain diseases, particularly its connection to COI. Foxo3a is known to be expressed in key brain regions like the hippocampus, frontal lobe, and occipital lobe, which play crucial roles in cognitive function (Braidy et al., 2015; Yuan et al., 2016; Cheng et al., 2018). In response to stress, intra-neuronal Foxo3a can activate or inhibit transcriptional regulation by shuttling between the nucleus and cytoplasm (Bahia et al., 2012). Furthermore, as a transcription factor, Foxo3a can also impact neuronal mitochondria, modulate mitochondrial DNA expression, boost mitochondrial adenosine triphosphate (ATP) production and COX activity, and improve mitochondrial function, all of which influence cognitive processes in the brain (Caballero-Caballero et al., 2013; Shi et al., 2016b). By coordinating various downstream signals such as autophagy, inflammation, mitochondrial function, oxidative stress, and apoptosis, Foxo3a has the ability to regulate neuronal behavior and ultimately impact cognition (Wong et al., 2013; Wang et al., 2021; Fu et al., 2023).

Foxo3a has an important role in neuronal response to external stimuli (Maiese et al., 2007; Polter et al., 2009). Individual behavioral changes significantly stimulate cortical Foxo3a activation (Zhou et al., 2012). In the acute immobilization stress (AIS) model, the expressions of peroxidase (Prx) III and Mn-SOD in neurons are upregulated by enhancing Foxo3a expression, forming an antioxidant defense (Jeong et al., 2011). In AD-like brain tissues, nuclear retention of Foxo3a is inversely correlated with DNA damage while positively associated with glutamine synthetase levels and cognitive repair efficacy (Fluteau et al., 2015). As a vital neuronal mediator (Orellana et al., 2023), Foxo3a is essential for the maintenance of neuronal survival. Knockdown of Foxo3a results in substantial neuronal apoptosis in embryonic zebrafish development (Peng et al., 2010). The level of Foxo3a expression varies as age in response to age-related brain damage (Sahin et al., 2013; Rollo et al., 2021). However, excessive activation of Foxo3a can lead to neuronal damage and COI. The regulatory role of Foxo3a on neurons varies significantly across different brain disease contexts. This review mainly discussed intricate relationship between Foxo3a and cognitive disorders, focusing on its effect on neuron.

## 2 The role of neuron in COI

**FIGURE 1:**
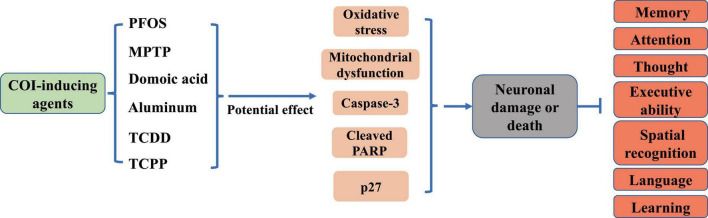
Effects of common COI-inducing agents on neurons and cognition.

Neuronal damage is the pathogenetic basis of COI in brain diseases (Figure 1). Elevated levels of neuronal death, inflammation, and oxidative stress have been observed in the hippocampal region of the brain in neurodegenerative conditions such as AD and PA (Ansari et al., 2024; Ramírez-Mendoza et al., 2024). The hippocampus, which is responsible for the storage and orientation of short-term memory, is one of the main anatomical structures involved in the formation of cognitive abilities. The hippocampal tissue mitochondria from AD individuals showed higher levels of reactive oxygen species (ROS), mitochondrial depolarization, reduced ATP, and calcium processing deficits (Olesen et al., 2024). Aging is a key trigger of cognitive deficits. Brain aging develops with age and is accompanied by increased levels of tissue inflammation, with neurons becoming progressively inflamed (Siddiqui et al., 2024). In an inflammatory milieu, activation of nuclear factor kappa B (NF-κB) and NLR family pyrin domain containing 3 (NLRP3) inflammasome leads to upregulation of pro-inflammatory cytokines such as tumor necrosis factor alpha (TNF-α), interleukin-6 (IL-6), and interleukin-1 beta (IL-1β), exacerbating neuronal apoptosis and worsening cognitive impairment (Jin et al., 2019; Liu et al., 2019). Furthermore, neuroinflammation results in detrimental outcomes including mitochondrial fragmentation (Harland et al., 2020) and endoplasmic reticulum stress (Huang et al., 2022). Various neurotoxins, such as perfluorooctane sulfonate (PFOS), 1-methyl-4-phenyl-1,2,3,6-tetrahydropyridine (MPTP), and domoic acid, can trigger neuronal damage (Gratuze et al., 2019; Wu et al., 2019; Petroff et al., 2022; Figure 1). In particular, aluminum, is known to induce severe cognitive deficits through persistent oxidative stress, increased N6-methyladenosine modification of brain-derived neurotrophic factor mRNA, and promotion of neuronal apoptosis (Song et al., 2024; Figure 1). Additionally, exposure to 2,3,7,8-Tetrachlorodibenzo-p-dioxin (TCDD) has been linked to cerebral and cognitive impairments, with TCDD activating caspase-3 and leading to extensive neuronal apoptosis (Xu et al., 2014; Figure 1). Recent research has shown that chronic exposure to environmental contaminants tris (1-chloro-2-propyl) phosphate (TCPP) can initiate abnormal neuronal oxidative stress and mitochondrial dysfunction, causing significant memory and cognitive deficits in zebrafish (Xia et al., 2021; Figure 1). Notably, depletion of dopamine (DA) neurons disrupts spatial learning, spatial memory, and object memory capacities (Morgan et al., 2015; Figure 1). These findings indicated that neuronal damage plays a pivotal role in the development of COI.

## 3 The feature of Foxo3a

**FIGURE 2:**
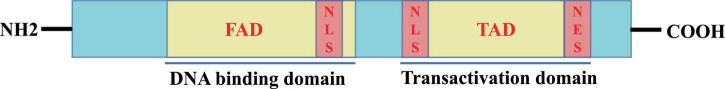
Schematic representation of the active structural domain of Foxo3a protein.

The Foxo3a gene is located on the human chromosome 6q21 and spans a total of 124,950 bases. The Foxo3a protein is comprised of 673 amino acids, with a molecular weight of 71,227 Da, displaying a distinct structural regularity (Figure 2). The core of the Foxo3a protein structure is featured by a highly conserved N-terminal Forkhead-associated domain (FAD) and a C-terminal Transactivation domain (TAD). The FAD is responsible for DNA interactions and binding, while the TAD serves as the fundamental structural domain for the transactivation of target genes by Foxo3a. Bidirectional movement between the nucleus and the cytoplasm is necessary for Foxo3a to exert transcriptional activation or repression activity. To fulfill this localization requirement, Foxo3a proteins typically contain two nuclear localization signal domains (NLSs) and one nuclear export signal domain (NESs).

**FIGURE 3:**
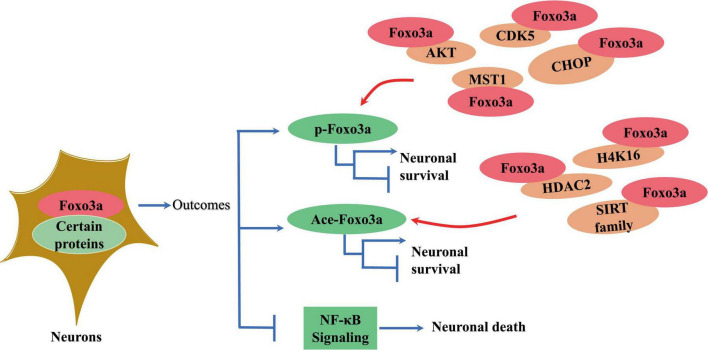
The effect of interaction of neuronal Foxo3a with other proteins on Foxo3a and neurons.

Foxo3a binds to gene sequences containing 5′-TAAAA-3′ to exert its transcriptional effects, playing a key role in regulating various cellular processes such as differentiation and apoptosis (Li et al., 2006; Ruankham et al., 2023). For instance, Foxo3a interacts with specific sequences in the promoter region of the pro-apoptotic protein Bim, impacting cell survival (Carbajo-Pescador et al., 2013). Notably, Foxo3a directly regulates transcription of mitochondrial DNA (mtDNA) to mediate mitochondrial respiration (Peserico et al., 2013). The transcriptional regulatory activity of Foxo3a requires the formation of polymeric complexes with multiple proteins (Figure 3). For example, the STAT3/Foxo3a signaling pathway has been found to mitigate excessive autophagy in neuronal PC12 cells (Xu et al., 2023; Figure 3). It has been reported that STAT3 cooperates with 14-3-3 to engage with Foxo3a, thereby sequestering Foxo3a within the cytosol and repressing its activity (Oh et al., 2012). Active aggregates centered on Foxo3a play a critical role in the transcriptional regulation of autophagy genes and the dynamics of autophagy (Xu et al., 2023). Inhibition of Foxo3a is a crucial step for ApoE to inhibit autophagy in the brain (Sohn et al., 2021). Additionally, Foxo3a phosphorylates or acetylates itself by binding to other proteins to regulate its intracellular localization (Figure 3). In cerebellar granule neurons, Foxo3a forms a complex with histone deacetylase 2 (HDAC2), which modulates P21 transcriptional expression and affects apoptosis induced by oxidative stress (Peng et al., 2015). Intriguingly, the interaction between Foxo3a and HDAC2 regulates the acetylation level of histone H4K16 in the p21 promoter region (Peng et al., 2015; Figure 3). SIRT3 serves as a classic acetylation repressor of Foxo3a. It has been suggested that SIRT3 may be involved in deacetylating Foxo3a to regulate Pink1/Parkin-associated mitochondrial autophagy in the brain (Wei et al., 2023). Thus, the interaction of Foxo3a with various factors is a critical step in modulating intracellular location. Besides, Foxo3a can also bind other proteins to alter their activity. It has been demonstrated that Foxo3a binds NF-κB to inhibit its nuclear translocation and reduces the exacerbation of inflammation caused by cerebral ischemia (Tan et al., 2021; Figure 3).

## 4 Regulation of neuronal Foxo3a

### 4.1 Methylation of promoter regions

The level of methylation in the promoter region of Foxo3a directly correlates with its expression. In cases of ischemia-induced neuronal apoptosis, there is a notable increase in methylation of the Foxo3a gene, leading to a significant suppression of its expression (Meng et al., 2022). Methyl CpG-binding protein 2 (MeCP2) is recruited to the promoter region of neuronal Foxo3a to enhance methylation levels (Meng et al., 2022). MeCP2 is recognized for its robust affinity for methylated DNA and its role in neurons has been extensively investigated. Mutant MeCP2 has been shown to disrupt functional connectivity among cortical regions in adult mice (Rahn et al., 2023). In neuronal cells, MeCP2 requires binding to TCF20 for proper function (Zhou et al., 2022). Dysregulation of TCF20 has also been linked to neurogenesis defects in the mouse cortex (Feng et al., 2020).

### 4.2 Phosphorylation

**FIGURE 4:**
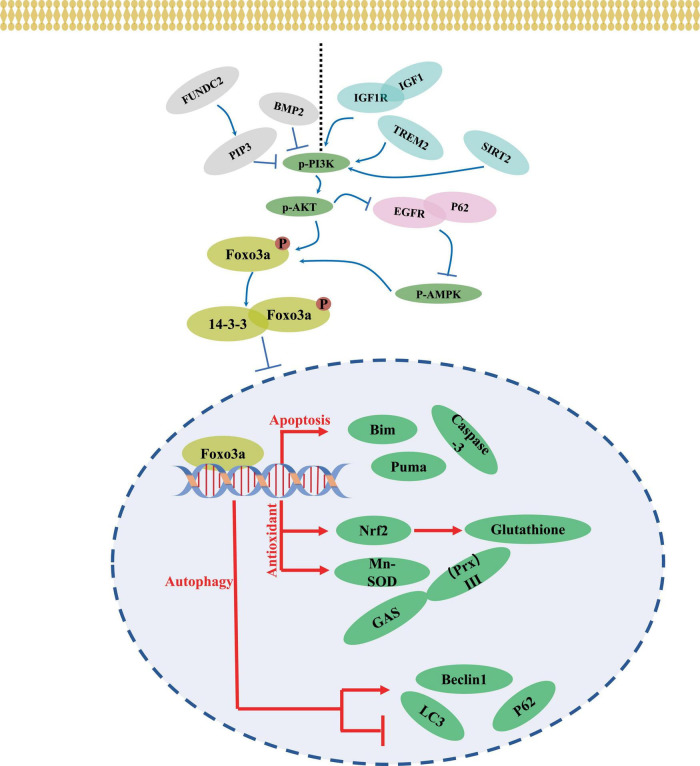
A mechanism of neuronal regulation through Foxo3a.

The transcriptional activity and subcellular localization of the Foxo3a protein are contingent upon various forms of modification, generally including phosphorylation, acetylation, methylation, and ubiquitination (Wang et al., 2017). Phosphorylation, a predominant inhibitory mechanism for Foxo3a and typically occurs at sites such as Thr-32 (Liu H. et al., 2014), Ser-7 (Ho et al., 2012), and Ser-253 (Li et al., 2006). Upon phosphorylation by the upstream kinase Akt, Foxo3a interacts with 14-3-3 proteins (Sanphui and Biswas, 2013), forming a complex that sequesters it in the cytosol, maintaining an inactive state. In neural-like PC12 cells, attenuation of FOXO3 phosphorylation and reduced Foxo3a recruitment to the promoter region of the pro-apoptotic protein bim were observed following Akt activation and ubiquitination (Xu et al., 2017). Treatment with thapsigargin, an endoplasmic reticulum (ERS) inducer (Figure 3), led to a marked decrease in Thr-32 phosphorylation of Foxo3a and an elevated nuclear-to-cytoplasmic Foxo3a ratio in neuroblastoma cells (Zhu et al., 2004). The diverse activities of the same protein resulting from phosphorylation at distinct sites by various phosphatases may yield contrasting outcomes. Nevertheless, Foxo3a phosphorylation does not invariably signify inactivation and is intricately linked to the interacting protein. For instance, Foxo3a can be phosphorylated and translocated to the nucleus to mediate neuronal death induced by oligomeric β-amyloid (Aβ) through mammalian sterile 20-like kinase 1 (MST1) (Sanphui and Biswas, 2013; Figure 3). The pro-transcriptional function of Foxo3a phosphorylated by Cdk5 has also been documented in neurons (Shi et al., 2016a; Figure 3). Conversely, Akt-mediated phosphorylation of Foxo3a represents a prevailing inhibitory mechanism in neuronal contexts (Wu et al., 2009; Zeng et al., 2017; Dong et al., 2018; Figure 4).

### 4.3 Acetylation

Acetylation constitutes a vital mechanism for the transcriptional activation of Foxo3a proteins. The Sirtuin (Sirt) family, renowned deacetylases, play a pivotal role in regulating the acetylation of neuronal Foxo3a (Figure 3). In neuroblastoma cells treated with ATRA, high expression of Sirt1 was accompanied by increased Foxo3a deacetylation (Kim et al., 2009). It was further found that in manganese-treated PC12 cells, activation of SIRT1 significantly inhibited the activity of Foxo3a (Zhao et al., 2019). However, acetylation also exerts an inhibitory effect on Foxo3a activation. Downregulation of Sirt1 expression was reported to significantly reduce Foxo3a activation in lidocaine-stimulated PC12 cells (Zheng et al., 2020). The concomitant high expression of Foxo3a and Sirt1 was detected in Parkinson’s-induced neuronal cells (Ubaid et al., 2022). Synergistic interaction of SIRT3 with Foxo3a is critical for the inhibition of hydrogen peroxide-induced oxidative stress in neurons (Liu et al., 2020; Ruankham et al., 2021; Yang et al., 2022). Sirt3-mediated deacetylation of Foxo3a regulates neuronal survival by the modulation of acetylation process (Mishra et al., 2018). Consequently, the functional and active state of SIRT-regulated Foxo3a acetylation is contingent upon the contextual conditions of treatment.

### 4.4 Upstream pathway of regulating neuronal Foxo3a

Numerous upstream pathways intricately intertwine with Foxo3a activity. AKT emerges as a ubiquitous regulator, exerting influence over Foxo3a through phosphorylation, thereby modulating its functionality. The PI3K/AKT axis has garnered attention for its role in orchestrating neuronal Foxo3a-mediated transcriptional regulation (Figure 4). Within the intricate network of the IGF1 signaling cascade, IGF1, IGF1R, and PI3K emerge as pivotal members. Notably, attenuation of IGF1R expression markedly diminishes AKT and Foxo3a phosphorylation within the murine brain (Liang et al., 2011). Intriguingly, the response of AKT/Foxo3a interplay to endoplasmic reticulum stress (ERS) is perturbed upon ablation of the ERS protein CHOP (Ghosh et al., 2012). Additionally, the upstream factor Triggering receptor expressed on myeloid cells 2 (TREM2) has been shown to potently activate Foxo3a within the AD brain tissues via the transduction of PI3K/AKT signaling (Wang Y. et al., 2020). In glioma stem cells, BMP4 stimulation elicits a decrement in p-AKT/p-Foxo3a signaling alongside an increase in the neuronal marker β-Tubulin III expression (Ciechomska et al., 2020). Moreover, the AMPK/Foxo3a axis exerts a pivotal role in governing autophagy within PC12 cells (He et al., 2022), with the AKT inhibitor SC66 demonstrating efficacy in impeding AMPK activation through modulation of the interaction between EGFR and autophagy protein P62 (Hou et al., 2022). Furthermore, heightened expression of FUN14 Domain Containing 2 (FUNDC2) promotes mitochondrial transport of phosphatidylinositol-3,4,5-trisphosphate (PIP3), which modulates neuronal AkT/Foxo3a signaling in a model of cerebral ischemia/reperfusion, consequently increasing the expression of bim (Shi et al., 2021). SGK1, as an AKT analog, has also been noted to synergize with AKT to mediate neuronal activation of Foxo3a (Sahin et al., 2013). It can be concluded that AKT, as a convergence point of the upstream pathway, has a profound impact on the neuronal cellular localization and functionality of Foxo3a (Sahin et al., 2013).

SIRT-centered signaling represents another pivotal upstream pathway influencing Foxo3a activity. Activation of SIRT3 leads to the deacetylation of Mn-superoxide dismutase (MnSOD), consequently elevating Foxo3a levels in ammonia-neurotoxic animal models (Anamika et al., 2023). Intriguingly, evidence suggests that SIRT1 activation may be dependent on PI3K/AKT signaling (Duan et al., 2019; Figure 4). Conversely, SIRT2 inhibitors AK1 and AGK2 have demonstrated the capacity to attenuate the p-AKT/Foxo3a signaling axis in ischemic stroke models (Duan et al., 2019). Simultaneous overexpression of SIRT1 and Foxo3a markedly mitigates oxidative stress induced by ischemia-reperfusion (Wu et al., 2020). However, during hypoxia-induced neuronal apoptosis, PARP1-dependent inhibition of NAD(+)/SIRT1 signaling promotes the acetylation and nuclear translocation of Foxo3a, and enhances the mRNA expression of Bcl-2/adenovirus E1B 19 kDa-interacting protein (Lu et al., 2014). Predictive E2F binding sites within the FOXO gene promoter region are noted. Moreover, the overexpression of E2F-1 increases the level of Foxo3a to modulate neuronal apoptosis (Nowak et al., 2007). In hepatocellular carcinoma cells, SIRT6 suppresses the transcriptional activity of E2F-1 (Ran et al., 2016), while the effect of this interaction on neuronal survival mediated by Foxo3a remains unexplored. Although SIRT-mediated deacetylation of neuronal Foxo3a has been extensively investigated, its effects necessitate contextual consideration within disease backgrounds and stimulus conditions.

MicroRNAs (miRNAs) are a class of non-coding single-stranded RNA molecules encoded by endogenous genes that regulate the post-transcriptional activity of genes. In recent years, miRNAs have also been extensively studied for their direct or indirect involvement in Foxo3a regulation of neurons. Such as miR-132/212, miRNA-7/211 have been observed to directly or indirectly regulate key molecules of the AKT signaling pathway, PTEN, Foxo3a as well as P300, to regulate the overall state of PI3K/AKT signaling and to affect the neuronal cellular activity (Wong et al., 2013; Salama et al., 2020). Phosphatase and tensin homolog (PTEN) inhibits the phosphorylation activation of neuronal Foxo3a by counteracting PI3K/AKT signaling (Zhao Y. et al., 2018; Zhao et al., 2021). Especially, the inhibition of PI3K/AKT/Foxo3a by miRNA-132 was shown to be an important aspect of sevoflurane-induced neuronal apoptosis (Dong et al., 2018). Additionally, miRNA-132 also affects the activity of Foxo3a by regulating PTEN (Zhao Y. et al., 2018). Several miRNAs such as miR-27a, miR-153-3p and miR-132-3p also directly bind to Foxo3a 3’UTR to manipulate its transcription in neurons (Sun et al., 2017; Wang H. et al., 2020; Fu et al., 2022).

## 5 Role of Foxo3a in cognitive disorders

Progressive COI associated with aging have emerged as a widely acknowledged concern. The full length Foxo3a isoform expression declined with age (Frankum et al., 2022). For example, Foxo3a expression is significantly down-regulated in the representative age-related disease intervertebral disc (IVD) degeneration (Alvarez-Garcia et al., 2017). The inhibition of PI3K/Akt signaling is accompanied by an increase of age-related renal Foxo3a level (Choi et al., 2012). The expression level of unphosphorylated Foxo3a was negatively correlated with increased age-related mortality (Rollo et al., 2021). SIRT1 is an acetylase of Foxo3a, and downregulation of its level causes significant acceleration of renal aging (Chuang et al., 2017). More importantly, Genetic inquiries have firmly linked mutated Foxo3a with aging and longevity (Brooks-Wilson, 2013). Especially, the rs2802292, rs2764264 and rs13217795 variants of FOXO3 have been associated with extreme longevity (Frankum et al., 2022). Investigations into Foxo3a’s involvement in aging-associated COI have also been undertaken (Baek et al., 2023). AD is a geriatric neurodegenerative disease characterized by representative COI. Clinical trials have shown that serum Foxo3a levels are significantly lower in AD patients compared to those with mild COI, suggesting its potential diagnostic utility in assessing AD risk (Pradhan et al., 2020). Cox proportional hazards modeling predicted that FOXO3 rs2802292 has the potential to inhibit the risk of the advance in AD in hypertensive patients (Chen R. et al., 2023). Possible mechanisms involve its reduction of blood-borne debris accumulation, increased oxidative stress, and inflammatory factor level in the brain due to hypertension-induced disruption of the blood-brain barrier (Chen R. et al., 2023). Further evidence suggests that inhibition of Foxo3a via PI3K/AKT exacerbates the onset of COI in AD mice (Wang Y. et al., 2020), mechanistically which may be related to the modulation of Foxo3a on mitochondrial autophagy for cognitive preservation (Zhou et al., 2024).

Foxo3a also exerts noticeable effects on COI that are not solely attributed to aging. In rats with severe cerebral ischemia/reperfusion-induced COI, a significant decrease in Foxo3a expression was observed, which was subsequently reversed after rehabilitation training (Jin et al., 2021). However, excessive expression of Foxo3a may also negatively impact cognition. TCDD, a common neurotoxin known to impede brain development and promote cognitive impairment, led to marked upregulation of Foxo3a expression in PC12 cells, accompanied by increased apoptosis (Xu et al., 2014). Elevated MST1 expression was detected in mice exhibiting impaired spatial memory, and inhibition of MST1 not only deactivated Foxo3a but also mitigated the memory deficits (Shang et al., 2020). PFOS, an environmental pollutant with widespread occurrence, has been linked to cognitive disorders. Suppression of Foxo3a prevented PFOS-induced apoptosis in PC12 cells (Wu et al., 2019). Excessive autophagy-induced damage to the hippocampus contributes to learning and memory deficits and represents an intrinsic factor underlying cognitive dysregulation in stroke (Gao et al., 2019). Abnormally elevated expression of Foxo3a has been observed in hippocampal neural stem cells affected by zinc deficiency (Han et al., 2015). Remarkably, in the aging brain, hippocampal neurons in the CA1 region exhibited heightened levels of active Foxo3a due to reduced Akt activity (Jackson et al., 2009). Antagonizing the PI3K/AKT/p-Foxo3a signaling cascade through interference of upstream PTEN results in enhanced pro-autophagic activation by Foxo3a, thereby promoting COI in stroke (Zhao et al., 2021).

Foxo3a is intricately connected with numerous upstream and downstream pathways. Various stimuli can induce diverse states and functions of Foxo3a, leading to activation of different downstream targets and thereby exerting distinct effects on neuronal states. Consequently, Foxo3a exhibits diverse effects on different types of COI. Transcriptionally, Foxo3a regulates the expression of several pro-apoptotic factors, including Puma and Bim, both of which are BH3-only pro-apoptotic proteins that synergistically induce neuronal apoptosis. Notably, treatment with Aβ has been shown to directly induce transcriptional regulation of Puma and Bim expression by Foxo3a (Akhter et al., 2014). Moreover, extrasynaptic NMDA receptors, known inducers of nuclear translocation of Foxo3a (Dick and Bading, 2010), have been implicated in neuronal apoptotic processes (Bahia et al., 2012). Foxo3a has also been implicated in mediating corticosterone-induced cell death in PC12 cells, which is closely associated with its activation of pro-apoptotic factor expression (Chang et al., 2023). Conversely, neuronal SIRT3/Foxo3a signaling is markedly upregulated in the presence of protective antioxidant enzymes such as Manganese porphyrins (MnPs) (Cheng et al., 2015). Notably, activation of Foxo3a enhances the expression of the antioxidant enzyme glutamine synthetase in the brain (Fluteau et al., 2015). In summary, Foxo3a modulates the effects of neuron-mediated cognitive alterations, with assessment necessitating integration of disease context and downstream pathways (Table 1).

**TABLE 1:** The role of Foxo3a in major models of COI was summarized.

Diseases	Model *in vivo* or *in vitro*	Downstream pathway	Biological functions	Potential effect on COI	References
AD	Apoe4 mice	Foxo3a upregulates the expressions of autophagy and mitophagy	Foxo3a ameliorates neuroinflammation	↑	Sohn et al., 2021; Zhou et al., 2024
	Aβ-stimulated neurons	Foxo3a upregulates expressions of bim, puma and caspases-3	Foxo3a induces apoptosis of neurons	↓	Sanphui and Biswas, 2013; Akhter et al., 2014; Saha and Biswas, 2015
Stroke	MCAO/R rats	Foxo3a alleviates mitochondrial oxidative damage through increasing MnSOD expression	Foxo3a inhibits neurological damage	↑	Xie et al., 2022
	MCAO/R rats	Foxo3a suppressed the expressions of BRCC3 and NLRP3	Foxo3a alleviates oxidative stress and neuroinflammatory responses	↑	Tan et al., 2024
	tMCAO rats	Foxo3a promotes the activity of CREB factor	Foxo3a attenuates neuroinflammatory responses	↑	Chen B. et al., 2023
IBI	Ovariectomized female rats	High expression of Foxo3a is accompanied by high level of caspase-3	Foxo3a induces apoptosis of neurons	↓	Jover-Mengual et al., 2010
	Hypoxia-ischemia rats	Foxo3a upregulates the expressions of bim and caspase-3	Foxo3a triggers apoptosis of neurons	↓	Li et al., 2009, 2013, 2017
	Cerebral I/R mice	Foxo3a promotes the ROS production	Foxo3a reduces apoptosis and excessive ERS	↑	Shi et al., 2019
	Cerebral I/R rats	Foxo3a suppressed the activation of nrf2/glutathione signaling pathway	Foxo3a potentially reduces neuronal survival	↓	Zaki et al., 2022
	Cerebral I/R rats	Foxo3a inhibits the activation of NF-κB signaling pathway	Foxo3a alleviates neuroinflammatory responses	↑	Tan et al., 2021
	OGD-stimulated neurons	Foxo3a upregulate the expression of bax, cleaved caspase 3, but reduces the level of bcl-2	Foxo3a protect against neuronal cell injury	↑	Meng et al., 2022
PA	Dopaminergic neuronal PC12 cells	Foxo3a activates the autophagy	No significant effect of Foxo3a on neuronal viability	Unknown	He et al., 2022
	MPTP mice	Increased level of Foxo3a is accompanied by increased level of MnSOD/NQO1/HO-1, but decreased NF-κB pathway	Foxo3a potentially rescues the loss of dopaminergic neurons	↑	Leem et al., 2024
	MPTP mice	Foxo3a upregulate the expression of bim	Foxo3a inhibits the apoptosis of neuron	↑	Liu L. et al., 2014
	6-hydroxy dopamine-treated cells and rats	Foxo3a promotes the expression of puma and FasL	Foxo3a triggers neuron death	↓	Sanphui et al., 2020; Bhattacharyya et al., 2023
TBI	Mice treated with weight drop	Acetylation of Foxo3a increased the level of bim	Acetylation of Foxo3a triggers apoptosis of neuron	↓	Xu et al., 2024
	Mice struck by convex tip	Foxo3a transcriptionally upregulates AQP4	Foxo3a leads to cytotoxic edema	↓	Kapoor et al., 2013
	Mice treated with weight drop	Foxo3a activates autophagy	Foxo3a initiates neuronal damage in the hippocampus	↓	Sun et al., 2018

MCAO/R, middle cerebral artery occlusion/reperfusion, tMCAO, transient middle cerebral artery occlusion, IBI, ischemic brain injury, OGD, oxygen-glucose deprivation, MPTP, methyl-4-phenyl-1,2,3,6-tetrahydropyridine, AQP4, Aquaporin 4. “↑” represents promotion of COI, “↓” represents inhibition of COI.

### 5.1 The downstream pathway of regulating AD by Foxo3a

**FIGURE 5:**
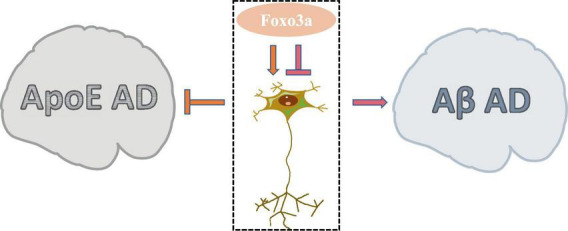
The dual role of Foxo3a in Alzheimer’s disease.

AD is a prevalent neurodegenerative condition affecting the elderly, characterized by COI. While the aggregation of Aβ and Tau proteins is commonly implicated in AD pathogenesis, the precise etiology of the disease remains elusive due to numerous contributing factors. Foxo3a, a protein extensively studied in the context of AD neurobiology, exhibits a dual modulatory role in neuronal regulation (Figure 5). The involvement of Foxo3a in AD pathophysiology is intricately linked to its interaction with specific disease triggers. Notably, alterations in these triggers can perturb Foxo3a-mediated regulation of AD neurological function.

Apolipoprotein E (ApoE) has been widely studied in AD due to its role in lipid and glucose metabolism, as well as its close interplay with neural signaling pathways. Dysregulated ApoE is recognized as a major risk factor for AD (Zhao N. et al., 2018), with ApoE4 being particularly implicated as the most potent pro-AD factor (Lanfranco et al., 2020). In AD brain tissues exhibiting elevated expression of ApoE4, there is a notable increase in the phosphorylation level of Ser253 on Foxo3a, accompanied by its suppressed activity (Sohn et al., 2021). The protection of Foxo3a against APOE-type AD potentially strongly is associated with autophagy. The disruption of autophagy exacerbate the progression of AD (Zhang et al., 2021), wherein the activation of Foxo3a exerts a pivotal role in promoting autophagic processes (Zhou et al., 2024). Moreover, emerging evidence demonstrated that Foxo3a mediates neurodegenerative pathways through the activation of the E3 ubiquitin ligase FBXO32/atrogin-1, thereby modulating autophagosome formation inhibition in AD.

Aβ, the primary constituent of cortical senile plaques, accrues with advancing age and represents a pivotal factor implicated in AD pathology. Aβ dimers have been shown to disrupt synaptic architecture and function, exerting neurotoxic effects. Notably, it has been demonstrated that treatment with Aβ upregulates neuronal Foxo3a expression, thereby directly facilitating the activation of downstream pro-apoptotic mediators including Bim, caspase-3, and PUMA (Sanphui and Biswas, 2013; Akhter et al., 2014; Saha and Biswas, 2015). Gonadotropin-releasing hormone (GnRH) exhibits notable anti-aging properties and exerts modulatory effects on neurodegeneration (Wang et al., 2010). GnRH has been documented to counteract Aβ-induced cytotoxicity (Marbouti et al., 2020). The modulation of hypothalamic on aging processes has been widely investigated (Kim and Choe, 2019). It has been elucidated that Aβ impedes GnRH expression in hypothalamic GnRH neurons by activating Foxo3a through NF-κB signaling pathways (Shi et al., 2020). Furthermore, Aβ-mediated dephosphorylation of Foxo3a in hippocampal neurons leads to its translocation to the mitochondrial nucleus and subsequent modulation of mtDNA expression, culminating in the suppression of cytochrome c oxidase subunit 1 (COX1) and ATP release, thereby precipitating mitochondrial dysfunction (Shi et al., 2016b). Attenuation of Foxo3a activation emerges as a promising therapeutic strategy for mitigating Aβ-associated AD pathology (Qin et al., 2008).

### 5.2 The downstream pathway of regulating stroke by Foxo3a

Stroke, characterized by the abrupt rupture of cerebral blood vessels or vascular obstruction leading to inadequate blood supply to the brain, results in the impairment of brain tissue. COI is a frequent complication after a stroke that can lead to disability, yet comprehensive and standardized rehabilitative strategies remain elusive (Cumming et al., 2013). The emergence of COI subsequent to stroke is attributed to structural damage within key brain regions including the temporal lobe, hippocampus, and brainstem. Neuronal damage within these regions is posited as a fundamental cellular mechanism underlying COI (Lipton, 1996). An augmentation of neuronal survival to ameliorate post-stroke COI represents a primary focus of contemporary research endeavors.

Ischemic and hemorrhagic strokes represent distinct pathophysiological entities. The overexpression of Foxo3a has been shown to impede the neurorestorative effects of artesunate following middle cerebral artery occlusion (MCAO) (Zhang et al., 2022), underscoring its role in exacerbating MCAO-induced neuronal injury, partly through dysregulation of autophagy (Xie et al., 2022). Conversely, activation of SIRT1 confers neuroprotection in MCAO/R rats, implicating the SIRT1/Foxo3a axis as a promising therapeutic target for MCAO (Tan et al., 2024). Furthermore, Foxo3a exerts influence in the context of hemorrhagic stroke. Activation of p-Foxo3a/CREB signaling contributes to the neuroprotective effects of Swell1 against cerebral ischemic stroke-induced neurological deficits (Chen B. et al., 2023), while phosphorylated Foxo3a impedes CREB nuclear translocation (Chen B. et al., 2023). Notably, CREB serves as a pivotal regulator of MeCP2 and DNMT3B methylases, with CREB-induced DNA hypermethylation identified as a contributing factor to susceptibility of ischemic stroke (Fan et al., 2023).

### 5.3 The downstream pathway of regulating IBI by Foxo3a

Ischemia induces neuronal apoptosis and disrupts cognitive performance. There is a significant positive correlation between ischemia-activated Foxo3a and neuronal apoptosis (Kuroki et al., 2009; Jover-Mengual et al., 2010; Li et al., 2013). In the early stage of ischemia and hypoxia, the levels of Foxo3a nuclear translocation, Bim and cleaved caspase 3 are significantly increased in brain tissue (Li et al., 2009, 2017). Enhanced oxidative stress activates the expression of mitochondria-associated pro-apoptotic proteins such as caspase 3 and Bax. Blockage of Foxo3a activation downregulates ischemia-induced increase of ROS (Shi et al., 2019). More importantly, it was found that inhibition of Foxo3a expression promotes the activation of the antioxidant pathway Nrf2/glutathione (Zaki et al., 2022). Further mechanistic studies revealed that increasing the methylation level of the Foxo3a promoter and inhibiting Foxo3a expression induces inhibition of the downstream SPRY2-ZEB1 axis and alleviates ischemic neuronal apoptosis (Meng et al., 2022). Additionally, Foxo3a could inhibit ischemia-induced neuroinflammation through direct interaction with NF-κB (Tan et al., 2021). Interestingly, activation of NF-κB was reported to potentially upregulate SPRY2 transcription (Day et al., 2020). Thus, ischemia is a crucial causative factor for COI.

### 5.4 The downstream pathway of regulating PA by Foxo3a

Parkinson’s disease is a neurodegenerative disorder characterized by the progressive loss of dopaminergic neurons in the midbrain, often accompanied by COI. Recent investigations in dopaminergic PC12 cells have illuminated a significant induction of autophagy following stimulation with the dopamine receptor (D1R) agonist SKF38393 (He et al., 2022). Further mechanistic exploration uncovered the regulatory role of upstream AMPK/ Foxo3a signaling in mediating D1R-dependent autophagy activation (He et al., 2022). In an MPTP-induced Parkinson’s model, the anti-inflammatory and antioxidative properties of creatine were found to be mediated by the activation of SIRT3/ Foxo3a signaling pathways (Leem et al., 2024). Moreover, additional studies unveiled the contribution of SIRT2 to MPTP-induced neuronal apoptosis through Foxo3a deacetylation and subsequent upregulation of Bim expression (Liu L. et al., 2014). The pro-apoptotic function of Foxo3a within Parkinson’s brain tissues, along with its downstream modulation of proteins such as Bim, PUMA, and FasL, underscores its role as a pivotal factor in Parkinson’s pathology (Sanphui et al., 2020; Wu et al., 2021; Bhattacharyya et al., 2023). Hence, Foxo3a emerges as a potential pro-Parkinsonian determinant.

### 5.5 The downstream pathway of regulating TBI by Foxo3a

TBI resulting from a direct impact or penetrating injury to the head damages brain tissue and affects brain function. Among the myriad complications ensuing TBI, COI stand prominent. Severe instances of TBI manifest a spectrum of cognitive discordances, encompassing visual and linguistic deficits, memory impairment, depressive tendencies, attentional lapses, and impediments in learning (Shuanglong et al., 2024). Notably, profound alterations in hippocampal morphology manifest within the traumatized cerebral milieu, concomitant with a marked decrement in neuronal density (Jahromi et al., 2024). Augmenting neuronal resilience constitutes the principal avenue toward ameliorating COI. Neuronal vitality and functionality are governed by an array of stressors, including oxidative insults and inflammatory cascades (Lu et al., 2024), with alterations in such responses contingent upon the orchestrated interplay of diverse signaling pathways.

Clinical investigations have unveiled a notable elevation in Foxo3a expression within traumatized human brain tissue compared to control group (Maiese et al., 2023). In animal models of traumatic brain TBI, the augmentation in Foxo-positive neuronal populations displays a temporal dependency (Liu et al., 2021). Mechanistically, acetylated Foxo3a was identified as a mediator driving Bim upregulation within traumatized cerebral tissue (Xu et al., 2024). Foxo3a was further delineated to directly stimulate Aquaporin 4 (AQP4) transcription, thereby promoting cytotoxic edema post-TBI and consequent memory deficits (Kapoor et al., 2013). Crucially, attenuation of Aquaporin 4 accumulation mitigates apoptosis and inflammation in TBI-afflicted brain cells (Xing et al., 2023). Additionally, Foxo3a exacerbated TBI-induced COI via activation of LC-3/p62-mediated autophagy (Sun et al., 2018; Liu et al., 2021). Notably, dysregulated autophagy emerges as a significant predisposing factor for compromised TBI recovery (Bao et al., 2016). Collectively, these findings underscore Foxo3a as a pivotal cytokine implicated in TBI pathogenesis.

## 6 Clinical prospective

Currently, rehabilitation evaluation stands as the principal approach for diagnosing COI. Concurrently, the advancement of technology has elevated imaging to a pivotal role as a cognitive assessment adjunct. Nonetheless, a notable deficiency persists in effective methodologies for evaluating COI from circulatory and pathological perspectives. The identification of a biomarker represents a pivotal advancement in the formulation of diagnostic modalities for cognitive disorders. Recent years have witnessed a significant increase in clinical trials and preclinical investigations, underscoring Foxo3a’s potential as a biomarker for diagnosing cognitive dysregulation.

Aging emerges as a focal risk factor for COI and notably serves as a core causative element in AD. A meta-analysis has underscored a significant association between Foxo3a rs2802292 and exceptional longevity (Revelas et al., 2018). Notably, serum assays conducted on AD patients unveiled markedly diminished serum Foxo3a concentrations compared to those observed in individuals with mild COI (Pradhan et al., 2020). Examination of human cortical tissue further revealed diminished Foxo3a levels in AD patients compared to non-AD counterparts (Sahin et al., 2013). Conversely, a heightened positivity rate for Foxo3a was observed in traumatized human brain tissue compared to non-traumatized specimens (Maiese et al., 2023). While these small-sample clinical studies hint at a potentially robust connection between *in vivo* Foxo3a alterations and human cognition, larger-scale cohort investigations remain imperative to bolster evidential support. Additionally, further validation is warranted to confirm the diagnostic precision of Foxo3a as a standalone marker for cognitive deficits. A comprehensive approach incorporating additional cognitive impairment biomarkers such as neurofilament light chain, various tau proteins, APOE, Aβ, and the autophagy marker molecule LC3 is essential when utilizing Foxo3a as a diagnostic tool. This integrative strategy is instrumental in enhancing diagnostic accuracy.

Currently, cognitive rehabilitation techniques serve as main treatment options of facilitating cognitive recovery, yet there remains a gap in pharmacological interventions. Foxo3a has emerged as a promising target for mitigating COI. Several studies have demonstrated the potential of clinical drugs that target the Foxo3a signaling pathway to effectively counter COI. Utilized in clinical settings, Chinese herbal medicines or compound formulas such as E. bonariensis, Chaigui granules, and Banxia Xiexin Decoction have exhibited neuroprotective effects by modulating Foxo3a expression in brain tissues, thereby enhancing cognitive function (Shi et al., 2023; Tian et al., 2023; Ibrahim et al., 2024). Additionally, the anti-malarial drug 8-aminoquinoline has demonstrated robust biological activity in improving neuronal mitochondrial function through the SIRT1/3-Foxo3a pathway (Ruankham et al., 2023). Perampanel, a widely prescribed antiepileptic medication, has shown efficacy in inhibiting neuronal damage following subarachnoid hemorrhage by targeting the SIRT3/Foxo3a signaling cascade (Yang et al., 2023). Furthermore, several candidate small molecules targeting neuronal Foxo3a signaling, including 3,14,19-triacetylandrographolide (Zhou et al., 2024), creatine (Leem et al., 2024), carboxy-terminal modulator protein (Miyawaki et al., 2009), estradiol (Jover-Mengual et al., 2010), and Ferulic acid (Picone et al., 2013), have surfaced. Nevertheless, their direct interaction with Foxo3a and specificity necessitate further comprehensive investigation to validate their selective targeting of Foxo3a.

Autophagy stands as a prominent downstream pathway in the regulation of Foxo3a within neurons, exerting a profound impact on cognition. The protective role of autophagy activated by Foxo3a in neurons is demonstrated by the upregulating expressions of ATG5, ATG7, ATG12 and LC3 (Wang et al., 2024). The activation of LC3 II and P62 are required for AMPK/Foxo3a-mediated autophagy machinery (Wan et al., 2020; Liang et al., 2021). Notably, Autophagy is a key protective response in the context of TBI. Inhibition of SKP2/CARM1 signaling by p-AMPK/p-Foxo3a activates autophagic flux in ischemic stroke mice (Zhao et al., 2023). Extensive research has elucidated the pivotal role of autophagy in neuronal function, particularly in the realm of cognitive repair (Li et al., 2023). When Foxo3a acted as an anti-COI factor, it significantly activates PINK1 and Parkin to enhance mitophagy, which in turn restores impaired mitochondria (Zhao et al., 2023). More importantly, upregulation of the autophagy signal BDNF/TrkB significantly attenuates the inflammatory response in hippocampal neurons (Gao et al., 2022). Overexpression of Foxo3a also autophagy flux. It has been highlighted that MAP1LC3B/LC3-associated phagocytosis is closely linked to amyloid β clearance (Lee et al., 2019). Consistently, autophagy enhancement promotes the degradation of Tau protein, thereby alleviating Tauopathy-related neuroinflammation and synapse loss to restore cognition (Zhu et al., 2023). Importantly, mitophagy maintains a balanced mitochondrial homeostasis via removal of the accumulated ROS and toxic fragments.

Spermidine, characterized by its indirect modulation of autophagy and potent antioxidant properties, exhibits the capacity to attenuate brain aging by bolstering neuronal autophagic processes (Xu et al., 2020; Yang et al., 2024). Encouraging findings from a single-center, randomized, double-blind, placebo-controlled Phase IIb trial underscore the efficacy of Spermidine in enhancing various neurocognitive faculties, including behavior and memory (Wirth et al., 2019). Moreover, Spermidine-enriched botanical extracts have demonstrated significant promise in mitigating cognitive decline among older individuals, with favorable safety profiles (Schwarz et al., 2018; Wirth et al., 2018). Importantly, 1.2 mg/day dose supplementation of Spermidine for 3 months had a favorable safety and tolerability profile. Furthermore, lithium has emerged as a facilitator of neuroplasticity through its modulation of autophagy pathways, as supported by clinical observations indicating enhanced hippocampal function following prolonged treatment (Forlenza et al., 2014). A 150 mg/day dose of lithium for 3 months was observed to have lower side effects, but the safety of long-term clinical use of lithium remains to be evaluated in specialized clinical trials (Forlenza et al., 2014). Notably, docosahexaenoic acid (DHA), an essential polyunsaturated fatty acid renowned for its diverse biological activities encompassing anti-aging effects, lipid modulation, and facilitation of brain development, etc. Administration of a 2 g/day dose of DHA over a 24-month period has been shown to elevate brain DHA levels, thereby correlating with enhanced memory, improved learning capabilities, and a potential preventive effect against AD (Pontifex et al., 2018). Several rigorous randomized, controlled clinical trials have corroborated the cognitive benefits of heightened DHA intake among the elderly, mechanistically implicating Aβ-mediated autophagic processes (Yurko-Mauro et al., 2010; Zhang et al., 2017, 2018). Despite the potential of Spermidine and DHA to combat cognitive impairment, there is still a need to consider the limitations of this dietary supplementation, pending more recent pharmacokinetic studies to comprehensively assess the effectiveness of these interventions.

## 7 Conclusion

Given the yet unclear elucidation of the mechanism underlying COI, there remains a notable absence of a reliable biomarker for its clinical diagnosis. Neurons, serving as the principal cellular constituency of the nervous system, intricately interconnect via synapses to construct elaborate neural networks forming neural circuits. These networks, in turn, orchestrate diverse cognitive functions encompassing memory, learning, and motion, etc. Notably, neuronal apoptosis and dysfunction emerge as pivotal endogenous factors precipitating COI observed in conditions such as AD, stroke, PA, IBI, and TBI. Thus, enhancing neuronal viability and functionality represents a critical frontier in the endeavor to remediate COI.

Foxo3a, functioning as a transcription factor, exerts direct or indirect influence over the activation and transmission of multiple pathways. Foxo3a demonstrated favorable improvement of neuronal activity and function in Apoe4 mice, MCAO/R rats, tMCAO rats, Cerebral I/R mice, Cerebral I/R rats, and MPTP mice as a potential protective factor. Conversely, its activity in Aβ-stimulated neurons, ovariectomized female rats, hypoxia-ischemia rats, and mice struck by convex tip demonstrated significant promotion of neuronal injury. Within the domain of neuron-mediated cognitive dysregulation, mitochondria-associated apoptosis protein including bim, puma and caspases-3, antioxidant pathway Nrf2 signaling and autophagy-related molecules including LC3, p62 and Beclin1, etc, stand out as principal downstream pathways regulated by Foxo3a. The modulation of Foxo3a itself is subject to various modifications, including promoter methylation, protein phosphorylation, and acetylation. Additionally, upstream pathways or molecules impacting Foxo3a encompass PI3K/AKT, SIRT, and micro-RNA. Notably, Foxo3a assumes diverse roles contingent upon the specific disease context within neuron-mediated COI.

Clinical observations suggested the potential of Foxo3a as a diagnostic marker for COI, particularly in AD. In particular, clinical evidence suggests that AD patients have lower levels of Foxo3a in serum and cortical tissues than non-AD or mild AD patients. However, immunohistochemical staining suggests that brain tissue from TBI patients expresses higher levels of Foxo3a compared to non-TBI populations, and these preliminary clinical trials demonstrate the potential of Foxo3a as a biomarker for diagnosing cognitive impairment. Several candidate small molecules have exhibited promise in clinical trials by targeting Foxo3a to facilitate cognitive repair. Notably, compounds such as spermidine, lithium, and DHA, acting upon the downstream autophagic pathway of Foxo3a, have demonstrated effectiveness in ameliorating cognitive disorders among afflicted patients. Additionally, multi-targeted therapy combining the neurofilament light chain, various tau proteins, APOE, Aβ, and the autophagy molecule may be able to improve the effectiveness of these drugs, which needs to be supported by more experiments in the future. More importantly, in-depth investigation and assessment are warranted in future studies to examine the dosage, frequency, side effects evaluation, pharmacokinetics, and precise efficacy of these medications. Large-scale clinical trials are imperative for determining the long-term effectiveness of these drugs. Consequently, elucidating the role of Foxo3a in the process of neuron-mediated cognitive repair warrants diligent investigation.

## Author contributions

Q-QL: Visualization, Writing – original draft, Writing – review & editing. G-HW: Writing – original draft. X-CW: Writing – original draft. X-WX: Writing – review & editing. R-W: Writing – review & editing. B-LY: Conceptualization, Writing – original draft, Writing – review & editing.
